# Heterotopic Ossification of the Adductor Muscles in Bald Eagles (*Haliaeetus leucocephalus*)

**DOI:** 10.3390/vetsci11110556

**Published:** 2024-11-10

**Authors:** Marko Legler, Katja von Dörnberg, Peter Wohlsein, Michael Fehr

**Affiliations:** 1 Department of Small Mammal, Reptile and Avian Medicine and Surgery, University of Veterinary Medicine Hannover, Bünteweg 9, 30559 Hanover, Germany; 2 Hanover Adventure Zoo, Adenauerallee 3, 30175 Hanover, Germany; 3 Department of Pathology, University of Veterinary Medicine Hannover, Bünteweg 17, 30559 Hanover, Germany

**Keywords:** bird of prey, muscular disease, myositis ossificans, raptors

## Abstract

Diseases of the musculature can be caused, for example, by alimentary or traumatic issues. In three bald eagles with painful lameness and/or a reduced range of the hip joint, a muscular disease was diagnosed via radiographic examination. The eagles were kept in a falconry and trained as demonstration birds, which were displayed on a gloved hand and flown in falconry education programs. The imaging revealed varying degrees of calcification of the adductors along the course of the puboischiofemoral muscle of the left hind limb in all cases. A histological examination of the affected musculature of one eagle confirmed the presence of a myopathy with muscle fiber atrophy, fibrosis, and metaplastic bone formation. Increased mechanical stress and injuries to the affected muscle groups are discussed as the cause of these alterations. The adductor muscles of the left leg of bald eagles in falconry husbandry appear to be particularly prone to muscular injuries and the development of heterotopic ossification. The healing process of such an injury with the stiffened musculature can lead to restrictions in the movement of the hip joint. Thus, the conscious and professional handling of the eagles is essential for maintaining the health of these birds.

## Simple Summary

Diseases of the musculature are not frequently described in birds of prey. In three case reports of bald eagles (*Haliaeetus leucocephalus*) with impairment of the function of the hip joint heterotopic ossifications of the adductors (*Myositis ossificans circumscripta*) were diagnosed. The eagles were kept in different falconries as demonstration birds. The diagnostic possibilities of this myopathy are discussed, along with imaging methods, possible therapy, and prevention.

## 1. Introduction

The bald eagle (*Haliaeetus leucocephalus*) is a large and heavy-bodied raptor weighing between 2.5 and 6.3 kg (females are up to 28% heavier than males). This eagle has a natural distribution on large bodies of water in North America. Fish comprise about 50 to 90 percent of the diet of this opportunistic feeder [1]. In Europe, bald eagles are mainly used in falconry as a display bird, either placed in exhibitions or trained for demonstrations where they are displayed on a gloved hand and flown in front of people [2]. Furthermore, these eagles are kept in suitable aviaries for breeding without the need for daily handling [2]. Little is found in the literature on muscular diseases in raptors [3,4,5]. In birds, capture myopathy, characterized by muscle tissue damage caused by complex physiological changes that involve metabolic acidosis due to elevated levels of lactic acid due to anaerobic glycolysis, is frequently described as a response to intense muscular activity [6].

Heterotopic ossification is defined as the formation of extra-skeletal bone in muscle and soft tissues and is a diverse pathological process [7,8]. However, in most cases of heterotopic ossification, it can be assumed that an aberrant tissue repair process is the main cause. A tissue injury is followed by an influx of inflammatory cells and the onset of a signaling cascade leads to metaplasia of mesenchymal cells, and thus to the development of osteoblasts and bone formation [8]. Heterotopic ossification in the musculature, also known as *Myositis ossificans*, is a commonly described complication of trauma and surgery in human medicine [7,8,9]. It is also described in dogs [10,11], horses [12] and other animals, for example, a vampire bat [13] and a kangaroo [14].

The aim of this case series is to describe the heterotopic ossification of the adductor musculature of bald eagles kept for falconry.

## 2. Materials and Methods

Three cases of bald eagles with muscular diseases of the adductors are presented in the following. The eagles in these cases were captive-bred, not directly related to each other, and came from different owners. All birds were kept for falconry and trained as demonstration birds, to be displayed on a gloved hand and flown in falconry education programs. The animals were fixed temporarily to jesses on the gloved hand, up to four times a day, and perches. The jesses are leather straps that are fastened around the raptors’ lower legs above their feet, allowing the falconer to secure the birds [2]. The jesses are essential for handling and for tethering the raptor to the perch [2]. The diagnosed muscular disease was, in one eagle (case 1), the main reason for being presented to the consultant in the clinic. In the two other birds (cases 2 and 3), the muscle disease was a secondary finding. In addition to the clinical examination, the birds were examined radiographically (cases 1, 2 and 3; GIERTH HF 400A High Frequency Diagnostik X-Ray Unit, GIERTH X-Ray International GmbH, Riesa, Germany; DX-D40G AGFA HealthCare, Bonn, Germany) and computed tomographically (cases 1 and 2; Brilliance 64, Philips Medical Systems, Best, The Netherlands). The blood chemistry values (dry chemistry analyzer; Table 1), and the red blood count (manual blood smear reviews; Table 1), were determined, after blood sampling from the *Vena Ulnaris*, in external laboratories (LABOKLIN, Bad Kissingen; SYNLAB.vet GmbH, Hamburg, Germany). The clinical examinations and imaging were performed under general inhalational mask anesthesia with isoflurane (Isofluran CP^®^, CP Pharma GmbH, Burgdorf, Germany), induced at 5% and maintained at 4 to 3%.

The musculature of the eagle of case 1 was examined histologically. For this purpose, tissue samples were fixed in 10% neutral-buffered formalin and embedded in paraffin wax according to a standard laboratory procedure. Sections of 3 µm thickness were cut and stained with hematoxylin-eosin and Azan stains, respectively.

## 3. Case Presentations

### 3.1. Case 1

**Figure 1:**
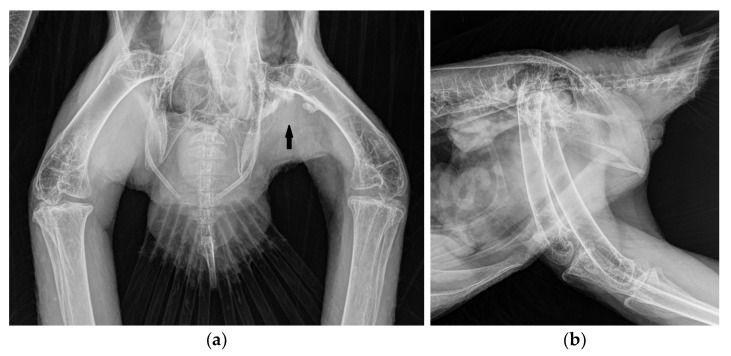
Ventro-dorsal (**a**) and latero-lateral (**b**) radiographs of the bald eagle of case 1 with heterotopic bone formations with a rough surface between the femur and pelvis, along the course of the adductors (arrow).

**Figure 2:**
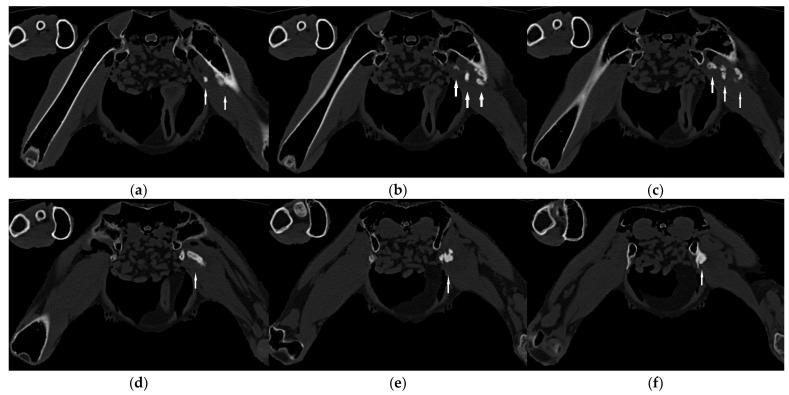
Axial computed tomographic images from the cranial (**a**) to caudal (**f**) area of the bald eagle of case 1 at the level of the hip joint (**a**–**f**). Heterotopic ossifications in the adductors (arrows) with a fusion in the pelvis (**d**) and the femur (**a**,**b**) and the partially existing eggshell appearance of the calcifications (**b**–**d**) are visible.

**Figure 3:**
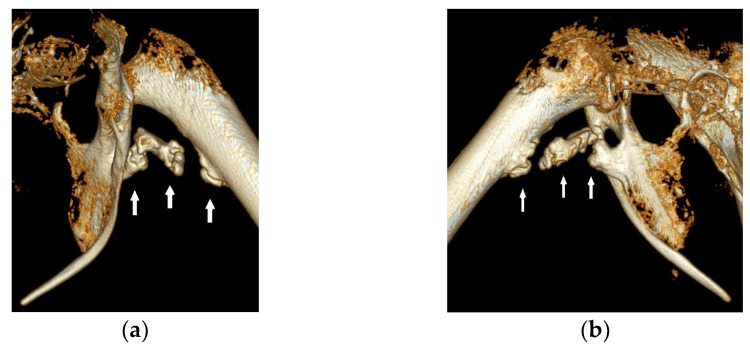
Cranial (**a**) and caudal (**b**) view of 3D computed tomographic reconstruction of the heterotopic bone formations (arrows) of the adductors of the bald eagle of case 1.

A one-year-old male bald eagle in training for a falconry program was presented with a two-week history of lameness in the left hind limb. The bird was in good body condition and showed undisturbed general health. Clinical examination revealed the limited mobility of the left hip joint, with swollen and hardened adductor muscles in the left leg. Under anesthesia, the affected leg was not fully extendable. The orthogonal radiographic projection of the pelvis and legs revealed calcifications along the course of the left adductor muscles (Figure 1). The edge of the affected areas appeared roughened and consisted of three fragments (Figure 1). CT scans confirmed the calcifications in the musculature with rough surfaces and revealed more details. The distal fragment was attached to the femur, while the proximal fragment was connected to the pubic bone and the ischium. A larger middle fragment was isolated in the musculature along the puboischiofemoral muscle (Figure 2 and Figure 3), and some of the fragments exhibited an eggshell appearance, with the interior of the calcification appearing less radiopaque than the exterior (Figure 2b–d).

The blood chemistry examination and the blood cell count revealed no abnormalities. The muscle enzymes, especially creatine-kinase (CK), aspartate-aminotransferase (AST), and lactate-dehydrogenase (LDH), were within the reference values for bald eagles (Table 1; [15,16,17,18]).

Conservative therapy was performed. The eagle was treated with non-steroidal anti-inflammatories (meloxicam 0.5 mg/kg BID for seven days, followed by SID for three weeks; Metacam^®^, Boehringer Ingelheim Vetmedica GmbH, Ingelheim/Rhein, Germany), antibiotics (doxycycline 50 mg/kg SID, for 7 days; Ronaxan^®^, Boehringer Ingelheim Vetmedica GmbH, Ingelheim/Rhein, Germany), and antifungals (itraconazole 10 mg/kg SID for 28 days; Itraconazol-ratiopharm^®^ l00 mg Hartkapseln, Ratiopharm GmbH, Ulm, Germany). The drugs were administered orally with food. During handling, the fixing of the eagle to the jesses was avoided and the animal was housed freely in an aviary closed on all sides and open on the top. The perches in the aviary were covered with astroturf to protect the bird from pododermatitis. The symptoms improved considerably during the first few weeks, and after a total of eight weeks, the owner no longer noticed any signs of lameness.

**Figure 4:**
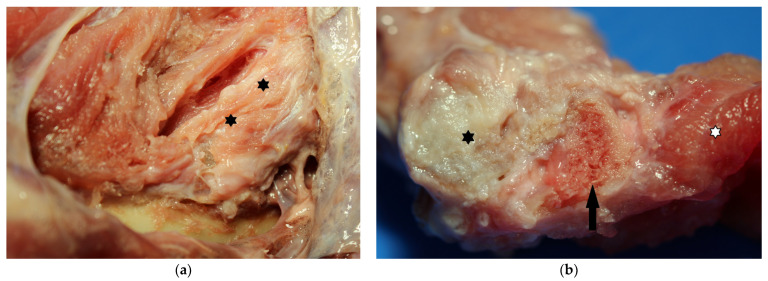
Pathomorphological examination: (**a**): strand-like hardening of the *M. puboischiofemoralis*, with a pale to whitish color (asterisks); (**b**): bone formations (arrow) in the diseased musculature (black asterisk); normal musculature (wight asterisk).

Unfortunately, after 18 months, the eagle died from an acute case of syrinx aspergillosis, and an examination of the diseased muscles was possible. A restriction in the mobility of the hip joint was still present, and the limb could not be fully extended. Pathomorphologically, a strand-like hardening of the *Musculus* (*M.*) *puboischiofemoralis* was detected. The altered tissue exhibited a pale to whitish color. Upon incision, bone formations were recognized in the musculature, confirming the imaging results (Figure 4).

**Figure 5:**
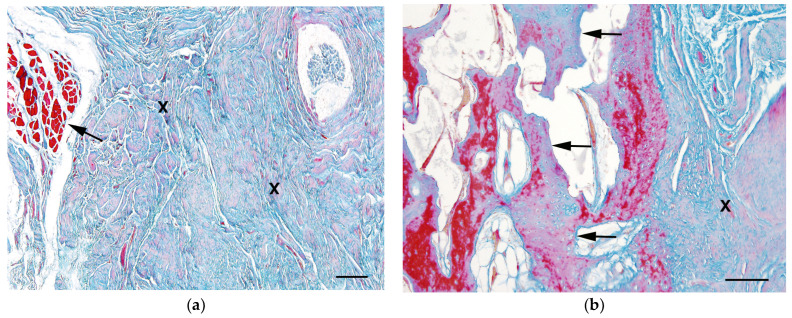
Histology revealed a severe loss of musculature with the replacement of collagenous fibrous tissue (X) with remnants of small muscle fibers (arrow)—Azan stain, bar = 200 µm (**a**); and a focally trabecular woven bone formation (arrows) is found with the fibrous connective tissue (X)—Azan stain, bar = 100 µm (**b**).

The histological examination of the altered musculature revealed a severe loss of musculature which had been replaced by collagen fiber-rich connective tissue and focal trabecular woven bone formations (Figure 5).

### 3.2. Case 2

**Figure 6:**
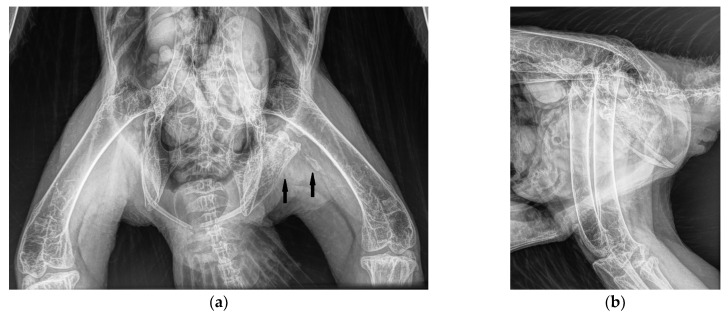
Ventro-dorsal (**a**) and latero-lateral (**b**) radiographs of a bald eagle (case 2) with heterotopic bone formations with a smooth surface between the femur and pelvis along the adductors (arrows).

**Figure 7:**
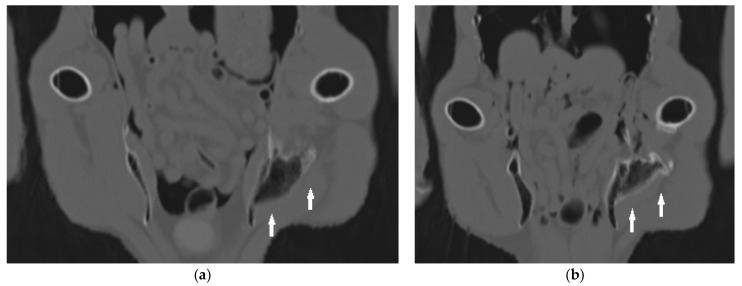
Horizontal computed tomographic images of a bald eagle (case 2) at the level of the pelvis and femur: more ventral (**a**) and more dorsal (**b**). Heterotopic ossifications in the course of the adductors (arrows) show fusion with the pelvis (**a**,**b**) and the femur (**b**). The large bone formation attached to the pelvis resembles a mature lamellar bone. In addition, the irregular structure of the musculature can be seen adjacent to the bone formations, especially in comparison to the healthy side.

An 18-year-old male bald eagle in training for a falconry program was presented for a health screening. The owner had not recognized any abnormalities in the past years. No clinical abnormalities were found in the examinations. However, the clinical examination under anesthesia revealed limited mobility of the left hip joint as a secondary finding. The left leg could not be fully extended. Orthogonal radiographic projection of the pelvis and legs and a CT scan revealed calcifications along the *M. puboischiofemoralis.* The calcification consisted of a larger area fused to the pelvis, and a small component fused to the femur. The edges of the calcifications were smooth (Figure 6 and Figure 7). A small area of calcification was also noted in the medial musculature of the femur. In addition, roughness was noted on the medial border of the femur. Blood chemistry and blood count values were within normal ranges (Table 1) [15,16,17,18]. No treatment of the muscular disorders was initiated in this case.

### 3.3. Case 3

**Figure 8:**
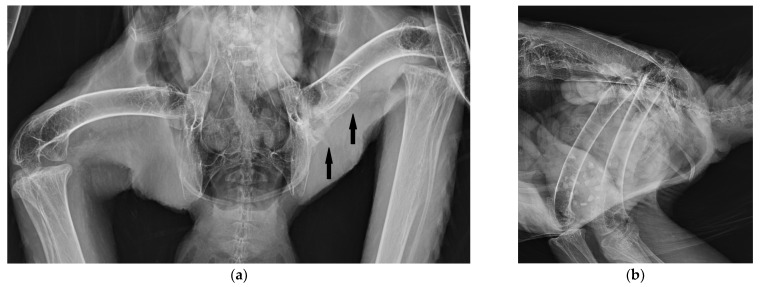
Ventro-dorsal (**a**) and latero-lateral (**b**) radiographs of a bald eagle (case 3) with heterotopic bone formations (arrows), with a smooth surface on almost the entire distance between pelvis and femur in the course of the adductors. The bone formations look like mature lamellar bone.

An 8-year-old female bald eagle was conspicuously had an injury to the right tarsal joint, with reduced function of the right foot, and performed weight bearing on the left while sparing the right leg. The eagle was also a demonstrating bird, flown in a falconry program. The examination of this eagle also revealed limited mobility of the left leg in the hip. Radiographs showed, as secondary findings, calcifications along the left *M. puboischiofemoralis.* Almost the entire distance between the pelvis and the femur was calcified. Two interruptions comparable to pseudojoints created a free middle section (Figure 8), and the bone formations resembled mature lamellar bone. The edges of the ossification were smooth and sharply defined. The adductor injury was also not treated in this case. The clinically unremarkable blood values are shown in Table 1 [15,16,17,18].

## 4. Discussion

**Figure 9:**
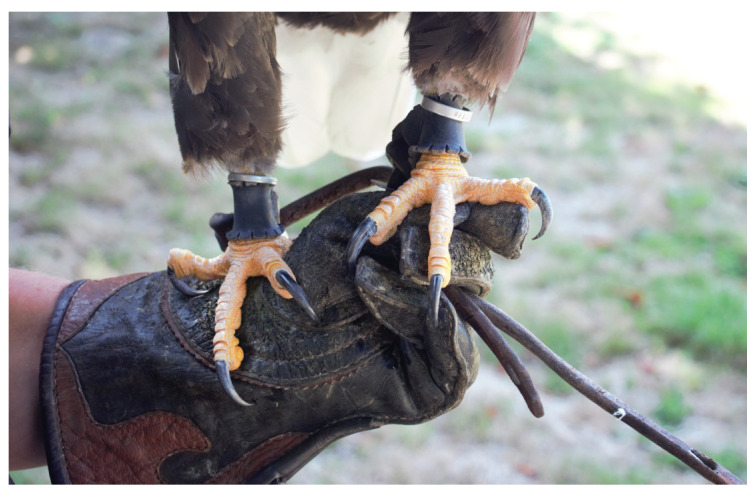
Illustration of an incorrect restraint of an eagle on the jesses of a gloved hand, which would result in a greater application of force on the left leg in a jumping-off situation.

**Figure 10:**
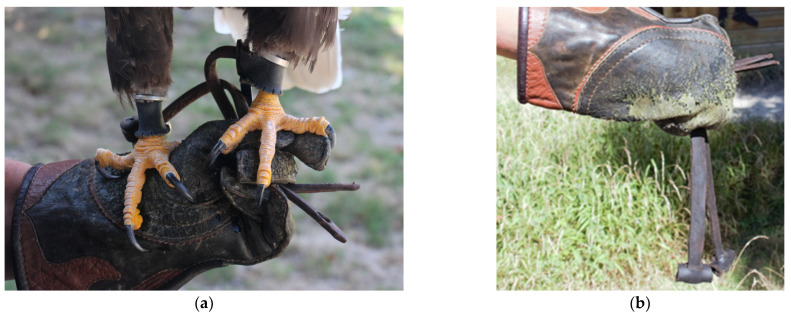
Illustration of the correct restraint of an eagle on the jesses of a gloved hand (**a**), with even weight distribution on both legs in preparation for a jumping-off situation (**b**).

The cases of the bald eagles in this study describe non-neoplastic self-limiting heterotopic ossifications in the adductor muscles, mainly along the puboischiofemoral muscle. To our knowledge, our case studies are the first descriptions of this muscle disease in eagles. Bald eagles of both sexes were affected. The eagles were of different ages and kept in comparable husbandry circumstances for falconry. Histological insights into the muscular disorder of one eagle revealed a loss of muscle structure, with formations of woven bone surrounded by connective tissue. Thus, the muscle changes of the dissected eagle showed a great similarity to the non-congenital heterotopic ossification (*Myositis ossificans circumscripta*) of human skeletal muscle [8]. One of the main causes of this metaplastic bone formation in human medicine is a recurrent local trauma with hematoma. A case of heterotopic ossification on a wing of a golden eagle (*Aquila chrysaetos*) associated with gunshot wounds shows that trauma and inflammation can be important for the development of this disease in eagles [19,20]. Muscle injuries could also play a decisive role in the etiology of the described muscle changes in the eagles in our cases. What is remarkable is that in all three eagles the left adductor musculature, in the course of the puboischiofemoralis muscle, was affected. This could indicate increased physical stress on this muscle group in eagles that were kept as demonstrating birds for falconry, in particular those affixed to jesses. A possible explanation for the increased physical stress on these muscle groups could be an incorrect temporary restraint. In practice, it is often observed that the outer jesses are kept shorter in order to restrict the range of the eagle (Figure 9). However, this incorrect restraint of a bird can lead to an uneven load of various forces on the muscles, compared to a proper restraint (Figure 10). If the eagle jumps off in this incorrect configuration, the bird’s entire body load pulls on the muscles of the left limb, especially on the adductors, and can cause an injury in this muscle group.

The heavy weight of an eagle, ranging from 2.5 to 6.3 kg, may also have a negative effect [2]. Daily handling of the eagles several times a day, affixed to jesses on the gloved hand, may cause repeated microtrauma to already damaged muscles, which favors the development of healing disorders. In human medicine, a premature return to activity after muscle damage is a primary risk factor for the development of *Myositis ossificans* [21]. Whether the keeping of the eagle in case 2 for falconry over a long period of time had an influence on the disease, or whether it was only a temporary single injury of the adductor muscles, cannot be comprehensively assessed. In addition, local hematomas can increase the risk of heterotopic ossifications, as seen in patients with coagulation disorders [22]. So far we have observed this muscle disease predominantly in bald eagles. One explanation for this observation could be the anatomy of bald eagles, which are mainly designed for catching small prey and not for such strong pulling forces. Thus, this eagle as a fish eater can be more sensitive to injuries than, for example, the golden eagle, which preys mainly on larger prey with stronger acting forces [23]. Evidence of a genetic cause of the muscle disease was not found in our cases; the eagles were not directly related [24].

Depending on the time after the injury of the muscles in humans, the clinical outcome can range from painful muscles with relief of the limb to a painless limb with possible limitation of limb movement [25,26]. These different disease patterns in humans also reflect our cases. In all of our cases, the stiffness of the affected muscles lead to an impairment of the function of the hip joint. In one eagle, the changes in the adductor muscles led to painful, restricted function of the limb. Compared to the other eagles, in this case, the disease is mainly characterized by bone formations with a rougher surface. This could indicate incompletely differentiated bone formation during a still active process. In case 2 and 3, the bone formation is more differentiated, the muscular remodeling process seems to be completed, and the eagles showed no signs of pain. The heterotopic ossification in skeletal musculature is, in human and small animal medicine, often described as self-limiting [27].

Obtaining a reliable diagnosis of this muscular disease is challenging. Using radiography and/or computer tomography, the calcified areas of the musculature can be visualized and a tentative diagnosis can be made. In particular, the eggshell appearance of the calcifications of the new bone provides a hint [7]. The characterization of the bony parts of the muscular changes seems to be useful to assess the degree of organization of this muscular disease. Important differential diagnoses along with calcifications in the musculature can be, for example, other causes of heterotopic ossifications due to circumscribed infections of the musculature or neoplastic diseases of the bones and muscles [7]. A biopsy can help in these cases with obtaining a final diagnosis [3,4,6]. However, as the dissection showed, the muscle lesions are not as circumscribed as it appears on images. There is also a risk of hemorrhage during the collection of the biopsy in a surgery. These hemorrhages can worsen the situation [7,21]. For this reason, we decided against such an intervention in our cases, especially since in two out of three cases it was an incidental finding that did not result in any problems for the eagles in daily life. Magnetic resonance tomography or a sonographic examination are also described in the literature to obtain a better characterization of the actual muscle damage and to detect early stages of hematoma and tissue edema in the initial first weeks after injury, independently of ossification [14,28]. In the advanced stages of our cases, the myopathy could not be detected by blood chemistry. In early stages, an increased CK level and a decreased Ca level can be indicative in the diagnosis [3,4,14,29].

There are no descriptions of the treatment of such a muscular disease in birds in the literature. Our cases show that the spontaneous self-limiting healing of lesions is possible in eagles. However, the formation of ossifications and connective tissue can affect the function of the musculature and the neighboring joints, as happened in our cases. All three eagles showed restricted mobility of the hip joint. Pain medication can help the animals to progress through the healing process better, as case 1 illustrates [4,30,31]. Presumably, the first signs of muscle trauma should lead to resting the eagles until they have fully healed in order to avoid aggravation. In our experience, healing muscle damage can take a minimum of two to four weeks, depending on the injury. This procedure is also recommended in human medicine [14,21,25,26]. In small animal and human medicine, surgical treatment with the removal of the altered tissue is described [9,32]. However, surgical intervention on the eagle at this point, medially on the femur, can also lead to a worsening of the situation due to further injuries. The visualization of the affected musculature from the medial side is rather more difficult in birds than in mammals [4]. Furthermore, the dissection in our first case shows that the changes in the musculature are not as well defined as the radiological examinations would suggest. Of course, prophylaxis with gentle handling of the animals should be the first priority. Proper fixation on equal-length jesses could help to prevent injuries through an even distribution of the load (Figure 10).

## 5. Conclusions

A non-neoplastic heterotopic ossification in the adductor muscles, mainly in the left puboischiofemoral muscle, can occur in bald eagles used in falconry, comparable to the *Myositis ossificans circumskripta* in human and small animal medicine. Obtaining a reliable diagnosis of this muscular disease is challenging. Using radiography and/or computer tomography, the calcified areas of the musculature can be visualized. Due to the eggshell appearance of the calcifications of the new bone in particular, a tentative diagnosis can be made. The characterization of the surface of the bony parts of the affected muscular seems to be useful to assess the degree of organization of this muscular disease. During the healing process in all our cases, a pain-free condition was reached, but the stiffness of the affected adductor muscles led to an impairment of the function of the hip joint. A possible cause of the heterotopic ossification of the adductor muscles of the bald eagles can be assumed to be an injury due to an incorrect fixation. Thus, conscious and professional handling of the eagles is essential for maintaining the health of these birds and a premature return to activity after a muscle damage should be avoided.

## Figures and Tables

**Table 1:** Blood chemistry values and the red blood count of the examined bald eagles.

Blood Values	Eagle Case 1	Eagle Case 2	Eagle Case 3
Alkaline phosphatase (AP; U/L)	-	84	-
Aspartate aminotransferase (AST; U/L)	183	317	278
Acetylcholinesterase (AChE; U/L)	2130	1510	1300
Glutamate dehydrogenase (GLDH; U/L)	-	<2	-
Lactate dehydrogenase (LDH; U/L)	406	163	149
Creatinine kinase (CK; U/L)	431	257	245
Alpha-amylase (U/L)	565	620	-
Cholesterol (mmol/L)	-	8.22	-
Triglyceride (mmol/L)	-	1.0	-
Uric acid (µmol/L)	331	431	240
Sodium (Na^+^; mmol/L)	141	152	-
Potassium (K^+^; mmol/L)	3.5	2.6	2.7
Calcium (Ca^+^^+^; mmol/L)	2.43	2.30	2.3
Phosphate (Phos; mmol/L)	0.80	0.76	0.50
Glucose (mmol/L)	-	16.61	-
Bile acids (BA; µmol/L)	32.23	44.46	8.7
Total protein (TP; g/L)	34.0	33.0	32.0
Albumin (Alb; g/L)	13.0	13.2	12.0
Leukocytes (cells/µL)	14.0	10.0	8.5
Heterophilic granulocytes (%)	55	47	45
Eosinophilic and basophilic granulocytes (%)	1	2	2
Lymphocytes (%)	39	48	49
Monocytes (%)	5	3	4
Hematocrit (%)	49	51	53

## Data Availability

Data are contained within the article.
